# Value of Urinary Neutrophil Gelatinase-Associated Lipocalin versus Conventional Biomarkers in Predicting Response to Treatment of Active Lupus Nephritis

**DOI:** 10.1155/2020/8855614

**Published:** 2020-10-07

**Authors:** Mohamed Abd El-Mohsen, Ahmed Tawfik, Walid Bichari, Sahar Shawky, Gamal Mady, Mohamed Hassan

**Affiliations:** Internal Medicine and Nephrology Department, Faculty of Medicine, Ain Shams University, Cairo, Egypt

## Abstract

**Introduction:**

Lupus nephritis (LN) affects almost two-thirds of systemic lupus erythematosus (SLE) patients. Despite initial aggressive therapy, up to 25% of patients with LN will progress to permanent renal damage. Conventional serum markers for LN lack the sensitivity of an ideal biomarker. Urinary neutrophil gelatinase-associated lipocalin (UNGAL) is an excellent biomarker for early diagnosis of acute kidney injury and predicting renal outcomes.

**Objective:**

To measure UNGAL among LN patients to correlate its levels with renal disease activity and to investigate its predictive performance in response to induction therapy. *Patients and Methods*. 40 SLE patients with biopsy-proven LN class III, IV, or V were randomly selected. The study was conducted in the internal medicine department and outpatient clinic in Ain Shams University Hospitals and completed after six months. UNGAL was measured at baseline, three-month follow-up, and after complete induction therapy.

**Results:**

In LN patients at baseline, the mean serum creatinine was 2.57 ± 0.96 mg/dL and the mean UNGAL was 33.50 ± 18.34 ng/dL. Mean UNGAL levels of complete response, partial response, and nonresponse groups were 14.48 ± 2.99 ng/mL, 34.49 ± 4.09 ng/mL, and 62.07 ± 14.44 ng/mL, respectively. Based on the ROC curve, we found a better performance of baseline UNGAL to discriminate the complete response group from partial and nonresponse groups to predict response to induction, outperforming conventional biomarkers. The area under the curve was 0.943, and the best cutoff level was 26.5 ng/mL (92.31% sensitivity and 88.89% specificity).

**Conclusion:**

UNGAL performed better than conventional biomarkers in predicting response to treatment of active LN.

## 1. Introduction

SLE is a challenging condition that has an unpredictable course that presents unique issues in diagnosis and management [1]. It mostly affects women during the childbearing period, up to 20% of cases being affected in childhood; it is characterized by loss of self-tolerance and development of autoantibodies (autoAbs) to nuclear self-antigens [2]. SLE diagnosis is based on characteristic clinical manifestations affecting joints, skin, central nervous system, and the kidneys along with serological markers [3]. The basis for diagnosis was established by the American College of Rheumatology (ACR) based on the presence of any four out of eleven criteria which were recently revised in 2015 [4]. LN is a major risk factor for morbidity and mortality in SLE, and 10% of patients with LN will develop end-stage renal disease (ESRD) [5]. Evaluation is straightforward and should include a urinalysis and measurement of kidney function, generally serum creatinine concentration or estimated glomerular filtration rate (eGFR) [6]. Renal biopsy is the gold standard in the diagnosis and classification of LN [7]. Renal biopsy, although considered to be a benign procedure, sometimes can have serious complications, even in expert hands, a reason why this cannot be performed repeatedly to assess renal status. Therefore, the goal of studying newer LN biomarkers is to overcome the drawbacks of the existing biomarkers and replace the need for renal biopsy [8]. Tubulointerstitial damage is important in predicting the progression of glomerular diseases and LN [9]. Neutrophil gelatinase-associated lipocalin (NGAL) is a 25-kD protein secreted by leukocytes and tubular epithelial cells under different conditions of stress or inflammation [10]. NGAL is widely expressed following ischemic or nephrotoxic injury in humans. NGAL is thought to mediate inflammatory responses by sequestering neutrophil chemoattractants such as leukotriene B4 and platelet-activating factor [11]. Several clinical studies found that UNGAL represented a very sensitive and highly predictive biomarker for progressive tubular and glomerular injury [12]. Furthermore, in a previous study on SLE patients, an increase in UNGAL levels correlated with renal disease activity [13]. In the current study, we hypothesized that urinary NGAL would significantly correlate with the severity of renal disease activity and might predict the renal response to induction therapy.

## 2. Objective

The objective of this study was to measure UNGAL among LN patients to correlate its levels with the severity of renal disease activity and investigate its predictive performance in the renal response to induction therapy.

### 2.1. Patients and Methods

#### 2.1.1. Study Population

Our study included 40 SLE patients with biopsy-proven LN class III, IV, or V. Patients fulfilled at least four of the ACR 1982 revised criteria to diagnose SLE. All patients were randomly selected from the internal medicine department and outpatient clinic in Ain Shams University Hospitals during 2018 and 2019. We excluded diabetic patients, patients on hemodialysis, renal transplant patients, and patients with nonlupus-related renal affection, drug-induced lupus, overlapping syndromes, urinary tract infection, active systemic infection, and active malignancies. All patients received standard induction therapy. Patients were divided into three groups based on the renal response to treatment. Patients with complete response were defined by the return of serum creatinine to the previous baseline plus a decline in urinary protein-to-creatinine ratio (UPCR) to <500 mg/g. Partial response was defined by stabilization or improved serum creatinine level plus a ≥50% decrease in UPCR. The nonresponse group of patients was defined by a sustained increase in serum creatinine level or a <50% decrease in UPCR.

#### 2.1.2. Treatment of Lupus Nephritis

Induction of remission for active LN was performed using a combination of pulse intravenous (IV) methylprednisolone and either pulse IV cyclophosphamide (CYC) or oral mycophenolate mofetil (MMF). Pulse IV methylprednisolone therapy regimen ranged from 500 to 1000 mg IV daily for three consecutive days. Six pulses of intravenous cyclophosphamide (0.5–1 g/m^2^) on consecutive months were offered to patients or MMF (2-3 g/d for six months) especially for women with childbearing potential. Maintenance dose of oral prednisolone 0.5–1 mg/kg per day was tapered slowly over a few weeks to the lowest effective dose.

#### 2.1.3. Clinical and Laboratory Measurement

All participants were subjected to detailed history taking, including sociodemographic history (age, sex, residence, and marital status), stressing on the presence or absence of constitutional symptoms (fever, malaise, myalgia, or weight loss), significant hair loss, skin rash, oral ulcers, and photosensitivity. An assessment was carried out for joint-, pulmonary-, cardiovascular-, renal-, hematological-, and neuropsychiatric-related complaints. Careful drug, menstrual, and family histories were taken. A thorough systemic physical examination was performed, including blood pressure measurement and skin and mucosal examination. Cardiac, rheumatological, and neurological examinations were performed. Assessment of disease activity by the SLE disease activity index (SLEDAI) scoring system [14] was carried out for each patient (the minimum score is 0 and the maximum is 105). Renal involvement was assessed by a renal SLEDAI (rSLEDAI) score of 4 (the minimum score is 0 and the maximum is 16), corresponding to the presence of any one of the following concerning urine analysis: hematuria, proteinuria, pyuria, or urinary red cell casts. Renal histological features were evaluated by a renal pathologist. Activity index scores were calculated from the summation of individual scores. The range of activity index score was 0 to 24 with higher scores representing higher activity. Chronicity index scores were calculated from the summation of individual scores. The range of chronicity index score was 0 to 12 with higher scores representing higher chronicity [15]. Blood samples were obtained at baseline, three months after induction, and after complete induction to determine complete blood cell count, serum creatinine level, blood urea nitrogen (BUN), C3 and C4 fractions of the complement, anti-nuclear antibodies (ANA), and anti-dsDNA antibody. Estimated glomerular filtration rate (eGFR) was assessed using the Cockcroft–Gault equation. Complete urinalysis and UPCR were performed.

#### 2.1.4. NGAL Enzyme-Linked Immunosorbent Assay

Urine samples were collected at baseline, three months after induction, and after complete induction, centrifuged at 2000–3000 rpm for approximately 20 minutes to remove particular impurities, and then stored frozen at −80°C until assayed. The UNGAL level was measured by the Biont human NGAL enzyme-linked immunosorbent assay (ELISA) kit (catalog no. YLA0724HU) which is a sandwich enzyme immune assay for in vitro qualitative measurement of NGAL. All measurements were made in triplicate and blinded manner. Urinary NGAL excretions were reported as the amount of urinary NGAL in nanograms per milliliter (ng/mL).

#### 2.1.5. Ethical Issues

The research followed the tenets of the Declaration of Helsinki. The research was approved by the Ethics Committee of Faculty of Medicine of Ain Shams University (FWA 000017585). Informed consents were obtained from all patients who participated in this study.

#### 2.1.6. Statistical Methods

Data were revised for its completeness and consistency. Double data entry was done on IBM SPSS Statistics version 23.0 (IBM Corp., Armonk, NY, USA). Quantitative data were summarized by mean along with standard deviation, while qualitative data were summarized by numbers and percentages. Student's *t*-test was used to compare quantitative data between two independent groups, and the one-way analysis of variance (ANOVA) test was used for more than two groups. A chi-square test was used to compare qualitative data between different groups. The repeated measures ANOVA test was used to compare quantitative data for the same group at different time points, and the Friedman test was used for qualitative data. Pearson's correlation test was used to measure the correlation between different continuous variables. Receiver operating characteristic (ROC) analysis was used to calculate the area under the curve (AUC) with associated 95% confidence interval (CI) for UNGAL and conventional biomarkers that were used to predict renal response and to find the best cutoff values to identify the renal response after induction therapy. A “*p* value” of less than 0.05 was considered statistically significant.

## 3. Results

A total of 40 patients were included in this study, most patients were females (87.5%), with a mean age of 25.63 ± 4.26 years. The duration of SLE ranged from recently diagnosed patients up to 10 years. About 20% of patients had constitutional symptoms of SLE. About 20% of patients had nephritis only, and the rest of the patients had systemic organ involvement in addition to nephritis. Disease activity that was assessed using the SLEDAI score was 19.55 ± 4.70. Renal activity assessed using the rSLEDAI score was 14.20 ± 3.13. Most of the patients were classified as LN class IV (45%). Coclassification with class V was found among nine patients (22.5%). Mean scores of activity index and chronicity index in biopsy were 7.53 ± 1.55 and 4.75 ± 1.21, respectively. At baseline, the mean UPCR was 2.89 ± 1.39 g/g creatinine, mean serum creatinine was 2.57 ± 0.96 mg/dL, and mean UNGAL was 33.50 ± 18.34 ng/mL. The patients' characteristics and laboratory results are described in Table 1.

### 3.1. Baseline UNGAL with Patients' Characteristics and Laboratory Results

Among LN patients, we found no relation between UNGAL with age, sex, duration of disease, systemic organ involvement, and different classes of LN. The UNGAL level correlated positively with rising blood pressure (systolic and diastolic) (*p*=0.01); moreover, positive correlations were observed between baseline UNGAL and each of the following: SLEDAI score (*p*=0.01), chronicity index (*p*=0.001), BUN, and serum creatinine (*p* < 0.001). UNGAL correlated negatively with complement C3 (*p*=0.01), while no correlations were found regarding UNGAL with proteinuria and rSLEDAI score; Table 2.

### 3.2. Patients' Characteristics and Baseline Laboratories in Different Groups (Classified according to Renal Response to Induction)

Patients received induction of remission, both clinical and laboratory parameters improved. We found a significant reduction in UNGAL levels after three months and after complete induction. Upon classifying the patients into three groups according to renal response to treatment, we found 13 patients with complete response, 19 patients with partial response, and eight patients with no response to induction therapy. Baseline UNGAL levels were significantly lower among patients with complete response (14.48 ± 2.99 ng/mL) than those with partial response (34.49 ± 4.09 ng/mL) and nonresponse (62.07 ± 14.44 ng/mL) (*p* < 0.001). We did not find a significant difference between the three groups as regards gender, age, disease duration, LN class, SLEDAI, and rSLEDAI scores. Significantly higher blood pressure (systolic and diastolic) was observed in the nonresponse group compared to the complete response group (*p*=0.03 and *p*=0.02). As expected, patients with no response had significantly higher renal chronicity index (*p* < 0.001), while there were no significant differences regarding the renal activity index. We found significantly higher proteinuria in patients with complete renal response compared to the partial response group of patients (*p*=0.002); moreover, patients with complete renal response had significantly lower serum creatinine and higher estimated GFR (*p* < 0.001). However, we found no significant differences in baseline complement levels among the three groups (Table 3).

### 3.3. Predictive Performance of Urine NGAL versus Conventional Biomarkers

**Figure 1:**
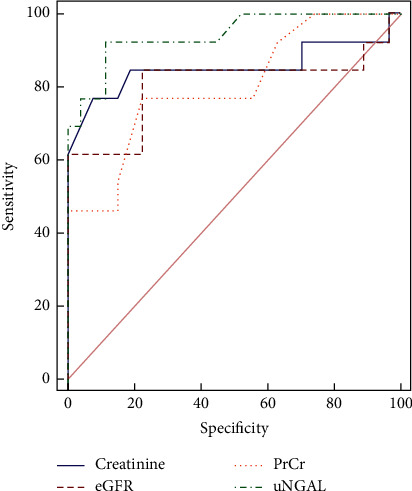
Graph ROC curves showing area under the curve of baseline UNGAL and conventional biomarkers to predict renal response to induction. It showed an AUC value of 0.943, outperforming conventional biomarkers.

**Figure 2:**
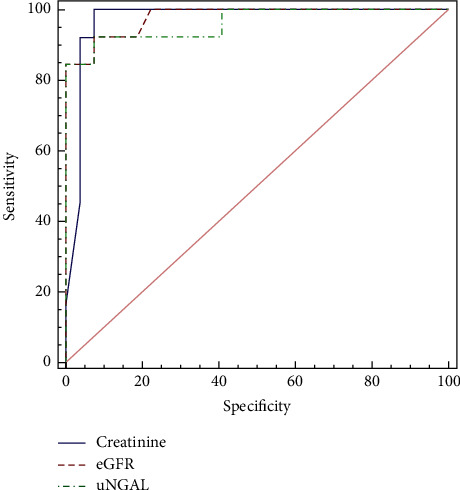
Graph ROC curves showing area under the curve of three-month follow-up UNGAL and conventional biomarkers to predict renal response to induction. It showed an AUC value of 0.966, with slight differences compared to other conventional biomarkers.

ROC analysis was performed to calculate the AUC with associated 95% CI comparing UNGAL and conventional biomarkers at baseline and three months after induction therapy, thus investigating the diagnostic reliability of UNGAL as a predictor of the renal response to induction therapy and differentiating the complete response group from partial and nonresponse groups. We found a better performance of baseline UNGAL to discriminate the complete response group from partial and nonresponse groups, outperforming conventional biomarkers. The area under the curve value was 0.943, and the best cutoff value was 26.5 ng/mL (sensitivity = 92.31% and specificity = 88.89%) (Tables 4 and 5; Figure 1). After three months, when compared to other conventional biomarkers, UNGAL was found with an area under the curve value of 0.966 versus creatinine with an area under the curve value of 0.966 and eGFR with an area under the curve value of 0.979. The best cutoff value for the three-month UNGAL was 17.4 ng/mL (sensitivity = 92.30% and specificity = 96.30%) (Tables 6 and 7; Figure 2).

## 4. Discussion

Biomarkers help in accurate evaluation of disease activity and enable the physician to individualize therapy. Biomarkers for LN should be different from the biomarkers of overall disease activity as LN requires significant immunosuppression. Consequently, prediction of renal activity and correlation with renal histological findings are needed in such biomarkers [8]. Despite the previous studies that were conducted to reveal the underlying mechanisms responsible for the pathogenesis of SLE, few biomarkers have been remarkably discovered. The lack of reliable biomarkers not only delays the clinical management of SLE but also delays the development of a newer therapeutic agent [16]. NGAL has emerged as a novel biomarker of great value for the diagnosis of kidney injury. NGAL is present in neutrophils, where it is bound with gelatin and usually expressed by the injured epithelia [17]. Previous studies have been conducted to evaluate the role of NGAL in the evaluation of kidney injury in patients with lupus nephritis [18, 19]. In the present study, we investigated the performance of UNGAL in predicting treatment outcomes compared to conventional biomarkers of LN disease activity. Our study results indicated that UNGAL might be a novel biomarker of LN. Findings from the present study indicate that urinary NGAL might be a biomarker of the renal response to induction therapy outperforming serum creatinine and other conventional biomarkers. Our study showed that baseline UNGAL did not correlate with age, gender, and systemic organ involvement which is in agreement with the study by Satirapoj et al. [20] and El Shahawy et al. [21]. As regards the relation between baseline UNGAL and ISN/RPS class, we found no significant differences in baseline UNGAL between different classes of LN which is in agreement with the study by Alharazy et al. [22]. Our study showed that baseline UNGAL correlated with disease activity as it correlated positively with rising blood pressure (systolic and diastolic) and negatively with C3 which is in agreement with the study by Satirapoj et al. [20]. We found positive correlations between baseline UNGAL and each of the following: chronicity index, SLEDAI score (which possibly might reflect the renal components of the SLEDAI score and worsening renal function), BUN, and serum creatinine. However, we found no correlation between baseline UNGAL and rSLEDAI score; a possible explanation might be that all patients in our study had nephritis proven by biopsy with almost comparable renal affection and rSLEDAI scores among complete, partial, and nonresponse groups. Our results agreed with those of Batool et al. [23] as regards the positive correlation between baseline UNGAL and serum creatinine, while we found no correlation between baseline UNGAL and proteinuria which is in contrast to the study by Satirapoj et al. [20]. In addition, the current study did not find significant correlations between baseline UNGAL and other laboratory parameters, including Hb, WBCs, platelets, and C4, which is in agreement with the study by El Shahawy et al. [21]. Upon classifying patients into three groups according to renal response to treatment to clarify the relationship between UNGAL and renal response, we found that baseline UNGAL levels were significantly lower among those with complete response than those with partial response with the highest baseline urine NGAL levels and those in the nonresponse group; these results agreed with those of El Shahawy et al. [21] and Satirapoj et al. [20], providing a strong evidence for UNGAL as a predictor for treatment response. As regards renal chronicity index in the biopsy, it was significantly higher in patients with no response compared to patients with complete response and partial response, while no significant differences regarding renal activity index among the three groups, this goes in hand with the study by Satirapoj et al. [20]. Surprisingly, regarding proteinuria, we found that patients with complete renal response had significantly higher proteinuria. This finding could be explained by the heavy proteinuria occurred in class V in the complete response group of patients. We found significantly lower serum creatinine and higher estimated GFR in patients with complete renal response, in contrast to the study by Satirapoj et al. [20]. Regarding baseline complement, we found no significant differences between the three groups, unlike the study by Satirapoj et al. [20]. Diagnostic reliability of the UNGAL in our study showed a good performance of UNGAL in predicting renal response to therapy, discriminating the complete response group from partial and nonresponse groups. As regards baseline UNGAL, ROC analysis showed an AUC value of 0.943 that was greater than those for other parameters (proteinuria, serum creatinine, and estimated GFR). Thus, it outperformed conventional biomarkers; the best cut-off value to discriminate the complete response group from partial and nonresponse groups was 26.5 ng/mL (sensitivity = 92.31% and specificity = 88.89%). These results mean that baseline UNGAL can be used with a lot of trust and with a very good predictive performance. Consequently, it may have the potential to predict poor response to induction therapy and perform better than conventional markers in predicting clinical response to treatment of active LN. We investigated the predictive performance of the three-month follow-up UNGAL in comparison to conventional biomarkers. We found better predictive performance of the three-month UNGAL compared to C3. While compared to other conventional biomarkers (serum creatinine and GFR), UNGAL was found with an area under the curve value of 0.966, with slight difference compared to other conventional biomarkers. The best cutoff value for the three-month UNGAL that discriminate the complete response group from partial and nonresponse groups was 17.4 ng/mL (sensitivity = 92.3% and specificity = 96.3%).

## 5. Conclusion

This study showed excellent diagnostic performance of UNGAL compared to conventional markers for predicting disease severity and clinical response to treatment of active LN so that it may become one of the most promising biomarkers in LN patients.

## 6. Limitations of the Study

Our study carries the limitation of a relatively short follow-up period of 6 months to demonstrate the further rise in serum creatinine or initiating long-term dialysis.

## Figures and Tables

**Table 1:** Patients' characteristics and laboratory results.

Variable	No. (%) or mean/SD
Female	35 (87.5%)
Age (years)	25.63 ± 4.26
Duration of SLE (months)	37 ± 29.5
Systolic blood pressure (mmHg)	130.00 ± 11.98
Diastolic blood pressure (mmHg)	75.00 ± 9.94
SLEDAI score	19.55 ± 4.70
rSLEDAI score	14.20 ± 3.13
*Systemic organ involvement*	
Nephritis only	8 (20.0%)
Arthritis	11 (27.5%)
Cutaneous lupus	11 (27.5%)
Serositis	11 (27.5%)
Hematological involvement	9 (22.5%)
Carditis	4 (10%)
Vasculitis	2 (5%)
Neurological lupus	2 (5%)
*ISN/RPS class*	
III	5 (12.5%)
IV	18 (45.0%)
V	4 (10.0%)
III + V	4 (10.0%)
IV + V	9 (22.5%)
Activity index in renal biopsy	7.53 ± 1.55
Chronicity index in renal biopsy	4.75 ± 1.21
*Immunosuppressive agents*	
IV cyclophosphamide	25 (62.5%)
Mycophenolate mofetil	15 (37.5%)
*Laboratory results*	
Serum albumin	2.56 ± 0.37 g/dL
Hemoglobin	9.89 ± 1.46 gm/dL
BUN	46.53 ± 8.86 mg/dL
Creatinine	2.57 ± 0.96 mg/dL
eGFR	41.63 ± 26.02 mL/min/1.73 m^2^
Complement component 3 (C3)	63.90 ± 19.65 mg/dL
Complement component 4 (C4)	16.75 ± 4.53 mg/dL
Pr/Cr ratio	2.89 ± 1.39 mg/mg
UNGAL	33.50 ± 18.34 ng/mL

SLE, systemic lupus erythematosus; SLEDAI, SLE disease activity index; rSLEDAI, renal SLE disease activity index; ISN/RPS, International Society of Nephrology/Renal Pathology Society; IV, intravenous; eGFR, estimated glomerular filtration rate; BUN, blood urea nitrogen; C3, complement component 3; C4, complement component 4; Pr/Cr ratio, protein-to-creatinine ratio; UNGAL, urinary neutrophil gelatinase-associated lipocalin.

**Table 2:** Correlation of baseline UNGAL with demographic, clinical, and laboratory data of patients.

Variable	UNGAL	
	*r* ^*∗*^	*p* value
Age	0.116	0.477
Duration of SLE	0.02	0.91
Systolic blood pressure	0.42	0.01
Diastolic blood pressure	0.43	0.01
SLEDAI score	0.40	0.01
rSLEDAI score	0.30	0.06
Activity index in biopsy	0.12	0.45
Chronicity index in biopsy	0.49	0.001
Serum albumin	0.23	0.15
Hemoglobin	−0.25	0.12
Baseline complement 3	−0.42	0.01
Baseline complement 4	−0.17	0.30
BUN	0.57	<0.001
Creatinine	0.72	<0.001
eGFR	−0.57	<0.001
Pr/Cr ratio	−0.29	0.07

^*∗*^Pearson's correlation: significant at 0.05. SLE, systemic lupus erythematosus; SLEDAI, SLE disease activity index; rSLEDAI, renal SLE disease activity index; eGFR, estimated glomerular filtration rate; BUN, blood urea nitrogen; Pr/Cr ratio, protein-to-creatinine ratio; UNGAL, urinary neutrophil gelatinase-associated lipocalin.

**Table 3:** Baseline characteristics and laboratory data of different groups (classified according to renal response to induction).

Variable	Complete response (*N* = 13)	Partial response (*N* = 19)	Nonresponse (*N* = 8)	F^*∗*^	*p* value
	Mean/SD	Mean/SD	Mean/SD		
Baseline SLEDAI	18.92 ± 4.21	18.74 ± 5.04	22.50 ± 3.82	2.090	0.14
Baseline rSLEDAI	13.54 ± 3.84	13.89 ± 3.09	16.00 ± .00	1.769	0.19
Activity index in biopsy	7.38 ± 1.45	7.47 ± 1.74	7.88 ± 1.36	0.257	0.78
Chronicity index in biopsy	4.08 ± 0.76	4.63 ± 1.01	6.13 ± 1.25	10.870	<0.001^bc^
Hemoglobin	10.62 ± 1.68 g/dL	9.37 ± 1.39 g/dL	9.95 ± 0.58 g/dL	3.131	0.06
Serum albumin	2.31 ± 0.37 g/dL	2.73 ± 0.32 g/dL	2.59 ± 0.24 g/dL	6.488	0.004^a^
C3	68.23 ± 24.93 mg/dL	64.58 ± 18.04 mg/dL	55.25 ± 11.26 mg/dL	1.108	0.34
C4	16.54 ± 5.46 mg/dL	17.05 ± 4.08 mg/dL	16.38 ± 4.47 mg/dL	0.080	0.92
BUN	38.54 ± 8.85 mg/dL	49.63 ± 6.17 mg/dL	52.13 ± 4.97 mg/dL	13.011	<0.001^ab^
Creatinine	1.76 ± 0.97 mg/dL	2.69 ± 0.57 mg/dL	3.57 ± 0.52 mg/dL	16.349	<0.001^abc^
eGFR	63.69 ± 36.07 mL/min/1.73 m^2^	32.95 ± 7.57 mL/min/1.73 m^2^	26.38 ± 4.37 mL/min/1.73 m^2^	10.607	<0.001^abc^
Pr/Cr ratio	3.95 ± 1.65 mg/mg	2.30 ± 0.96 mg/mg	2.57 ± 0.78 mg/mg	7.549	0.002^a^
UNGAL	14.48 ± 2.99 ng/mL	34.49 ± 4.09 ng/mL	62.07 ± 14.44 ng/mL	111.428	<0.001^abc^

^*∗*^One-way ANOVA test (post hoc test: ^a^complete response vs. partial response/^b^complete response vs. nonresponse/^c^partial response vs. nonresponse): significant at 0.05 level. SLE, systemic lupus erythematosus; SLEDAI, SLE disease activity index; rSLEDAI, renal SLE disease activity index; eGFR, estimated glomerular filtration rate; BUN, blood urea nitrogen; C3, complement component 3; C4, complement component 4; Pr/Cr ratio, protein-to-creatinine ratio; UNGAL, urinary neutrophil gelatinase-associated lipocalin.

**Table 4:** Comparison between complete responders with partial and nonresponders as regards to baseline renal biomarkers.

Variable	Complete responders (*N* = 13)	Partial and nonresponders (*N* = 27)	Test value^*∗*^	*p* value
	Mean ± SD	Mean ± SD		
C3	68.23 ± 24.93 mg/dL	61.81 ± 16.68 mg/dL	−0.966	0.340
C4	16.54 ± 5.46 mg/dL	16.85 ± 4.12 mg/dL	0.203	0.841
Creatinine	1.76 ± 0.97 mg/dL	2.95 ± 0.68 mg/dL	4.486	0.0001
eGFR	63.69 ± 36.07 mL/min/1.73 m^2^	31.00 ± 7.36 mL/min/1.73 m^2^	−4.575	0.0001
Pr/Cr ratio	3.95 ± 1.65 mg/mg	2.38 ± 0.91 mg/mg	−3.884	0.0001
UNGAL	17.35 ± 7.22 ng/mL	39.55 ± 16.28 ng/mL	4.675	0.0001

^*∗*^Student's *t*-test: significant at 0.05 level. C3, complement component 3; C4, complement component 4; eGFR, estimated glomerular filtration rate; Pr/Cr ratio, protein-to-creatinine ratio; UNGAL, urinary neutrophil gelatinase-associated lipocalin.

**Table 5:** Cutoff levels for baseline UNGAL and conventional biomarkers in predicting renal response to induction.

Variable	AUC	Cutoff point	Sensitivity	Specificity	PPV	NPV
Creatinine	0.853	1.9 mg/dL	76.92	92.59	83.3	89.3
eGFR	0.806	36 mL/min/1.73 m^2^	84.62	77.78	64.7	91.3
Pr/Cr ratio	0.802	3 mg/mg	76.92	77.78	62.5	87.5
UNGAL	0.943	26.5 ng/mL	92.31	88.89	80.0	96.0

eGFR, estimated glomerular filtration rate; Pr/Cr ratio, protein-to-creatinine ratio; UNGAL, urinary neutrophil gelatinase-associated lipocalin.

**Table 6:** Comparison between complete responders with partial and nonresponders as regards three-month follow-up renal biomarkers.

Variable	Complete responders (*N* = 13)	Partial and nonresponders (*N* = 27)	Test value ^*∗*^	*p* value
	Mean ± SD	Mean ± SD		
C3	101.15 ± 22.27 mg/dL	91.48 ± 18.66 mg/dL	−1.442	0.157
C4	26.15 ± 5.51 mg/dL	26.67 ± 5.22 mg/dL	0.286	0.776
Creatinine	1.12 ± 0.24 mg/dL	2.53 ± 0.81 mg/dL	6.107	0.0001
eGFR	80.15 ± 24.09 mL/min/1.73 m^2^	36.59 ± 10.63 mL/min/1.73 m^2^	−7.994	0.0001
Pr/Cr ratio	1.52 ± 0.68 mg/mg	1.38 ± 0.58 mg/mg	−0.701	0.487
UNGAL	13.38 ± 5.83 ng/mL	35.23 ± 13.47 ng/mL	5.576	0.0001

^*∗*^Student's *t*-test: significant at 0.05 level. C3, complement component 3; C4, complement component 4; eGFR, estimated glomerular filtration rate; Pr/Cr ratio, protein-to-creatinine ratio; UNGAL, urinary neutrophil gelatinase-associated lipocalin.

**Table 7:** Cutoff levels for three-month follow-up UNGAL and conventional biomarkers in predicting renal response to induction.

Variable	AUC	Cutoff point	Sensitivity	Specificity	PPV	NPV
Creatinine	0.966	1.3 mg/dL	92.31	96.3	92.3	96.3
eGFR	0.979	51 mL/min/1.73 m^2^	92.3	92.6	85.7	96.2
UNGAL	0.966	17.4 ng/mL	92.3	96.3	92.3	96.3

eGFR, estimated glomerular filtration rate; UNGAL, urinary neutrophil gelatinase-associated lipocalin.

## Data Availability

The data used to support the findings of this study are included within the article.
